# Lanka virus, a *Mus booduga*-borne orthohantavirus infection-associated febrile illness in Sri Lanka

**DOI:** 10.1371/journal.pntd.0013169

**Published:** 2025-06-11

**Authors:** Devinda S. Muthusinghe, Pavani Senarathne, Kenta Shimizu, Yomani D. Sarathkumara, Shanika Nanayakkara, Sithumini M.W. Lokupathirage, Zhouoxing Wei, Nipun S. Rathnayake, Rydhnieya Vijeyakumaran, Nobuo Koizumi, Tomonori Kawakami, Akio Koizumi, Kouji H. Harada, Nilanthi Dissanayake, Senanayake A. M. Kularathne, Yoshimi Tsuda, Shuzo Urata, Jiro Arikawa, Chandika D. Gamage, Kumiko Yoshimatsu

**Affiliations:** 1 Hokkaido University Graduate School of Infectious Diseases, Sapporo, Japan; 2 Hokkaido University Institute for Genetic Medicine, Sapporo, Japan; 3 University of Peradeniya Faculty of Medicine, Peradeniya, Sri Lanka; 4 Gunma University Graduate School of Medicine, Gunma, Japan; 5 Institute for Molecular Bioscience, The University of Queensland, Brisbane, Australia; 6 University of Sydney Faculty of Medicine and Health, School of Dentistry, Sydney, Australia; 7 National Institute of Infectious Diseases, Tokyo, Japan; 8 Toyama Prefectural University Faculty of Engineering, Toyama, Japan; 9 Kyoto University Graduate School of Medicine, Kyoto, Japan; 10 Nagasaki University National Research Center for the Control and Prevention of Infectious Diseases, Nagasaki, Japan; Public Health Agency of Canada, CANADA

## Abstract

**Background:**

In Sri Lanka, a high seroprevalence of antibodies against hantaviruses was reported in communities affected by chronic kidney disease of unknown etiology (CKDu). Recently, two rodent-borne hantaviruses, Lanka virus and Anjozorobe virus, were identified in these areas. However, it is unclear which virus is the source of infection in humans, and its pathogenicity is unknown.

**Methodology/principal findings:**

A total of 181 sera from febrile patients from two CKDu-endemic regions, Girandurukotte and Polonnaruwa, were examined and Lanka virus genome was detected in two IgM-positive febrile patients. Of 76 serum samples from patients with fever of unknown etiology collected during 2016 examined to identify hantavirus genomes, antibodies, and serotypes, 10 were IgG-positive with five of them having IgM also. They were all without clinical features of hemorrhagic fever with renal syndrome, but three patients required treatment in the intensive care unit. A serotyping strategy was established based on the antigenic difference of the glycoprotein Gn of Lanka and Anjozorobe viruses. Using this method, febrile patients were found to be infected with the Lanka virus and none of the patient sera showed Anjozorobe virus infection pattern. Additionally, a total of 373 previously diagnosed seropositive serum samples from CKDu patients and healthy residents were serotyped to categorize 87% of seropositives as Lanka virus infection.

**Conclusions/significance:**

Lanka virus carried by little Indian field mouse *(Mus booduga)* is transmitted to humans, likely causing febrile illness occasionally while leading to severe disease in some of the febrile patients.

## Author summary

Hantaviruses are known to be transmitted by rodents and cause severe diseases such as hemorrhagic fever with renal syndrome (HFRS) in humans. Although there have been few reports of typical HFRS in Sri Lanka, there are many antibody-positive individuals. The antibody positivity rate is particularly high in patients with chronic kidney disease by unknown etiology (CKDu), and the relationship between hantavirus infection and CKDu is being discussed. Meanwhile, rodent surveys have identified Lanka virus in little Indian field mouse *(Mus booduga*) and Anjozorobe virus in black rat (*Rattus rattus*) in Sri Lanka. However, it was unclear which virus was infecting the humans and its pathogenicity. This study showed that hantavirus infections in Sri Lanka could be asymptomatic or cause common fever-like symptoms and rarely require treatment in an ICU. It was also shown that the main source of infection is Lanka virus. This study leads us to the starting line of clarifying the Lanka virus-related health risks, such as its association with CKDu.

## Introduction

Hantaviruses are a group of zoonotic pathogens that belong to the family *Hantaviridae* of the order Bunyavirales. Human infections caused by hantaviruses have two forms: hemorrhagic fever with renal syndrome (HFRS), which is typically found in Eurasia, and hantavirus cardiopulmonary syndrome (HCPS), which is usually found in the Americas [1]. East Asia accounts for approximately 90% of HFRS cases caused by Old World orthohantaviruses, such as the Hantaan virus (HTNV) and Seoul virus (SEOV) [2]. Southeast Asia, South Asia, and the Indian Oceanic regions are home to the Thailand orthohantavirus (THAIV) [3] and its genetic variants, the Anjozorobe (ANJZV) [4], Serang [5], Jurong [1], and Mayotte [6] viruses. The pathogenicity of these viruses remains unknown owing to inadequate epidemiological data. Several seroepidemiological reports have described human infections involving THAIV in Thailand, India, and Sri Lanka [3,7–9] and ANJZV in Madagascar [10]. Epidemiological information on hantaviruses and their hosts is limited, particularly in South Asian countries [2].

Hantavirus infection in Sri Lanka was first documented in 1988 [11]. Since then, several studies have reported hantavirus infections in Sri Lanka. Muthugala *et al.* reported IgM positivity in suspected HFRS cases and observed pulmonary edema in some cases [12]. Atypical severe cases of hantavirus infection have also been reported by two different groups [13,14]. Seropositive cases have also been reported in hospitalized patients [15]. However, the causative hantavirus species were not identified in these reports.

Apart from these studies on acute-phase patients, the available literature describes the existence of THAIV or THAIV-related hantavirus infections among humans and rodents in chronic kidney disease of unknown etiology (CKDu)-endemic areas in Sri Lanka [16–21]. Among patients with CKDu in endemic areas, Girandurukotte and Polonnaruwa, reported a seropositivity rate of approximately 50%, whereas healthy residents reported a prevalence rate of 15–18%. In contrast, healthy residents in non-CKDu-endemic areas have a low seropositivity rate (3%) [18,19,22]. These observations indicate that there are many opportunities for hantavirus infection in Girandurukotte and Polonnaruwa. However, typical HFRS was not observed in these regions.

CKDu has been increasingly diagnosed in the dry zones of Sri Lanka and has become an overwhelming public health burden [23]. This disease has been observed in rural agricultural communities [24], where males are more often affected than females [25]. Approximately 20,000 patients with CKDu have been reported in the North-Central province alone, with a population prevalence rate of 4.7% [26]. Water quality in these hotspots is suspected to be related to CKDu [27]. However, despite the many studies conducted over the past few decades, the etiology of CKDu remains unclear.

Recently, we identified the genomes of two THAIV-related orthohantaviruses from rodents captured in Polonnaruwa, a CKDu-endemic area in the North-Central province of Sri Lanka [28]. A genome detected from *Rattus rattus* was identified as a variant of ANJZV. In contrast, five other genomes, all identified from little Indian field mouse (*Mus booduga*), named Lanka virus (LNKV) currently designated as a novel orthohantavirus species, *Orthohantavirus lankaense*. However, the effect of LNKV infection on humans has not been clarified yet.

In this study, a serotyping strategy for LNKV and ANJZV was established. Then febrile patient sera were screened in two CKDu-endemic regions in Sri Lanka, Girandurukotte and Polonnaruwa, using serological and molecular biological methods to investigate the pathogenic potential of LNKV and ANJZV in humans. In addition, sera from patients with fever of unknown etiology collected in 2016 were retrospectively examined. Finally, a total of 373 anti-hantavirus antibody-positive sera collected over a period of 10 years were retrospectively serotyped. According to the results of these approaches, LNKV is the main source of hantavirus infections, likely causing febrile illness without the clinical features of typical HFRS in Sri Lanka.

## Methods

### Ethics statement

Ethical approval for this study was obtained from the Institutional Ethical Review Committee (ERC), Faculty of Medicine, University of Peradeniya, Sri Lanka (Project No. 2015EC/SP/15, Project No. 2016/EC/64, and 2021/EC/44 for the National Research Council [NRC] study) and written consents from all participants were preserved in University of Peradeniya, Faculty of Medicine, Department of Microbiology. Ethical approval for 2010 sampling was approved from the ERC of Kyoto University, Japan (G0313-5) with oral consents from all participants. And the ERC of the Institute for Genetic Medicine, Hokkaido University, Japan (21-001) was approved to treat Sri Lankan human materials based on G0313-5, Project No. 2015EC/SP/15, Project No. 2016/EC/64, and 2021/EC/44.

### Development of serologic assays based on viral antigens

Since previous genetic evidence indicated the circulation of LNKV and ANJZV among rodent populations, these novel viral antigen-based assays were developed in this study to serologically diagnose and serotype these two viruses. The N protein coding regions of LNKV and ANJZV were amplified from infected rodent lung cDNA by PCR using primers listed in S2 Table. Amplified DNAs were cloned into a mammalian expression plasmid vector, pCAGGS/MCS to construct pCLNK-N and pCSA-N recombinant plasmids for LNKV and ANJZV respectively.

Similarly, the GP coding regions of both viruses were amplified by PCR using the primers listed on S2 Table and cloned. Plasmids containing the M segment ORFs of LNKV and ANJZV were designated pCLNK-M and pCSA-M, respectively. The Gn glycoprotein portion of the GP gene of the two viruses were also amplified as above using the primer pairs shown in S2 Table. The amplified Gn coding regions were cloned into pCAGGS/MCS to obtain pCLNK-Gn and pCSA-Gn recombinant plasmids for LNKV and ANJZV respectively. All the plasmids were verified by sequence analysis. All the primers used to prepare the plasmid constructs are listed in the S1 Table.

Next, the rNs and rGPs of LNKV and ANJZV were transiently expressed in BHK/T7-9 cells using the prepared respective plasmids and TransIT-LT1 transfection reagent (Mirus Bio, Madison, WI, USA) according to the manufacturer’s instructions. Antigenic profiling of the rGPs was performed using an indirect immunofluorescence antibody (IFA) assay with two different panels of mouse monoclonal antibodies raised against the GP of the prototype HTNV [29]. We also established an alternative neutralization assay by using rGPs of LNKV and ANJZV as previously described (36) and the details are shown in the S1 Text. However, a distinction between LNKV and ANJZV infections by the comparison of neutralizing antibody titers was not possible due to high cross-reactivities, necessitating the need for other strategies.

For the serotyping of human sera, recombinant glycoprotein Gn (rGn) antigens were transiently expressed in BHK/T7-9 cells using the respective plasmids and the same transfection protocol, followed by the preparation of IFA slides. Next, rGn-based IFA assay was performed using sera from homologous hantavirus-infected rodents [28] to verify the reactivities. Briefly, serially diluted serum samples were added to the antigens and incubated at room temperature (18–25 °C) for 2 hours. Bound antibodies were detected using Alexa Fluor 488-conjugated goat anti-rat IgG (H + L) (A21210; Invitrogen, Carlsbad, CA, USA) or anti-mouse IgG (H + L) (A11029; Invitrogen) at a 1:1000 dilution. Endpoint titers (EPs) were determined based on characteristic fluorescent patterns in the cell cytoplasm.

### Human samples

**Fig 1:**
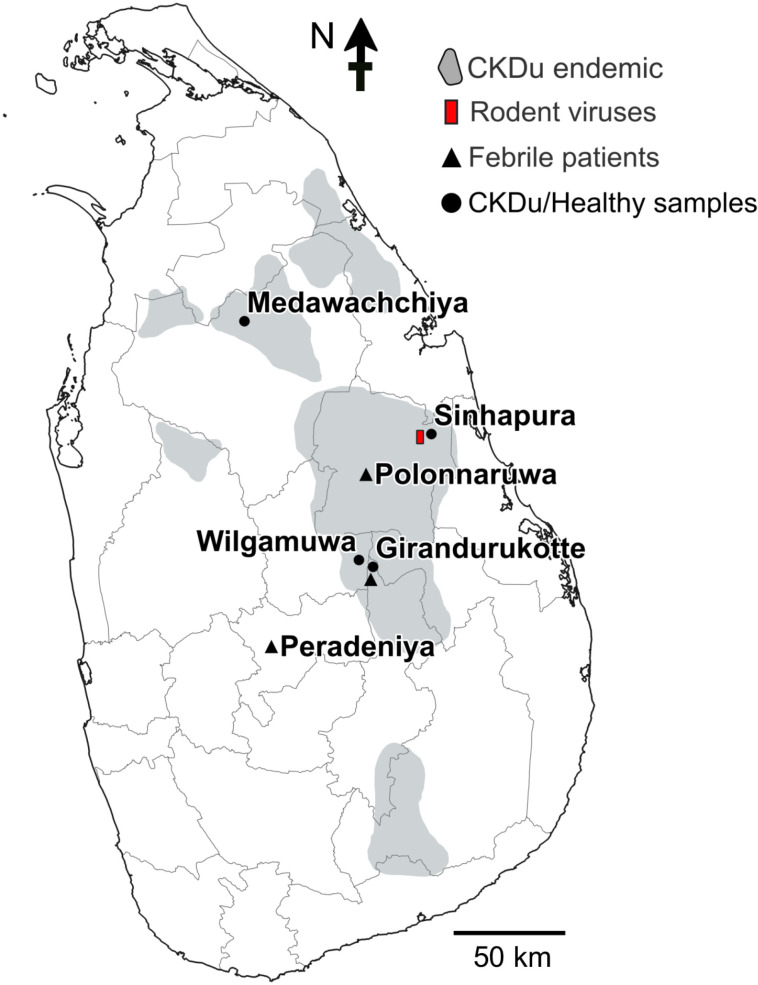
Map of Sri Lanka showing the areas of origin of human samples used for the study. The region where rodent-borne virus sequences used in this study were previously identified is also indicated. The basemap of Sri Lanka was obtained from the Database of Global Administrative Areas (https://gadm.org/download_country36.html) and is freely available for academic use.

Three groups of human blood and serum samples were used, as follows. The locations of the sampling areas are shown in Fig 1.

i) Febrile patients (n = 181) visiting the Polonnaruwa and Girandurukotte regional hospitals from January to October 2022 were recruited to collect blood samples to detect the presence of the hantavirus genome and anti-hantavirus antibodies. The inclusion criteria were individuals with acute fever who were registered in the outpatient departments of the divisional hospitals. Patients with suspected respiratory diseases, septicemia, acute wounds, or dengue fever confirmed, and those under the age of 18 years were excluded from this study.
ii) Serum samples of hospitalized acute febrile patients (n = 76) received from several hospitals at the Department of Microbiology, University of Peradeniya, in 2016 for the confirmatory diagnosis of hantavirus infection were also used for the study. These patients experienced episodes of acute febrile illness of unknown origin.
iii) Blood samples from patients with CKDu (n = 242) and healthy controls (n = 131) collected from several CKDu hotspot regions in Sri Lanka from 2010 to 2019 [16,18,30] were used for serotyping. Sera from both groups were previously confirmed to be positive for anti-hantavirus IgG antibodies [19].

### Serologic screening of samples from febrile patients

Anti-hantavirus IgM antibodies were detected in febrile patient sera using enzyme-linked immunosorbent assay (ELISA) with HTNV recombinant N protein (rN) antigens, as described [31]. IgG antibodies were detected using an IFA assay based on THAIV-infected and THAIV-rN-expressing Vero E6 cell antigens, as described previously [18].

### PCR screening of febrile patients

To detect the hantavirus genome, total RNA was extracted from patient plasma preserved in RNAlater (Invitrogen) using an QIAamp Viral RNA Mini Kit (Qiagen, Hilden, Germany) according to the manufacturer’s instructions, followed by cDNA synthesis using a ReverTra Ace qPCR RT mix (Toyobo, Osaka, Japan). All cDNA samples were screened via a hemi-nested PCR using degenerate primers targeting a conserved region of hantavirus L-genome [32]. The HAN-L-F2 (5'-TGCWGATGCHACIAARTGGTC-3') and HAN-L-R1 (5'-AACCADTCWGTYCCRTCATC-3') primers were used for the first round of PCR, followed by hemi-nested amplification using the HAN-L-F2 and HAN-L-R2 (5'-GCRTCRTCWGARTGRTGDGCAA-3') primers. Nucleotide sequences of the amplified PCR fragments were determined using the MinION platform (Oxford Nanopore Technologies Inc., Oxford, UK) using Ligation Sequencing Kit (SQK-LSK109) and Native Barcoding Kit (EXP-NDB104) according to the standard protocols. Partial fragments of the S and M segments were amplified using the primers listed in S1 Table. Nucleotide sequences were determined using a BigDye Terminator v3.1 cycle sequencing Kit (Applied Biosystems, Foster City, CA, USA) and a 3130xl Genetic Analyzer (Applied Biosystems). Sequences were manually edited and analyzed using Geneious Prime 2022.2.1 software (Biomatters Ltd., Auckland, New Zealand).

We assessed the phylogenetic relationship of the partial genome sequences obtained from febrile patients with other Muridae-borne hantavirus species, including the LNKV and ANJZV genomes detected previously from Sri Lankan rodents using Geneious Prime 2022.2.1 with the neighbor-joining method [33] employing the Tamura–Nei distance model [34].

### Serologic characterization and serotyping

Human serum samples suspected of acute hantavirus infection were subjected to IFA assays against different authentic and recombinant hantavirus antigens (i.e., THAIV-infected and/or PUUV-infected Vero E6 cell antigens), as well as the rN, rGP, and rGn antigens of LNKV and ANJZV. Experiments involving THAIV and PUUV infections to prepare the IFA antigens were performed at the BSL-3 facility of the Institute for Genetic Medicine, Hokkaido University. Serum samples with anti-GP antibodies were used for serotyping using the rGn-based serotyping IFA assay with modifications to the serum incubation time and temperature (human sera were incubated at 4 °C for 16 hours with rGn antigens). A four-fold difference in end-point titers against the two rGn antigens was considered positive for the selected virus type.

IgG-positive serum samples from all the groups were selected for serotyping after screening with rGP-based IFA assay. Anti-rGP antibody-positive samples were then serially diluted and applied for the rGn-based serotyping IFA assay, as described above. All the IFA assays were checked by two to three independent individuals.

Data availability the authors confirm that all data underlying the findings are fully available without restriction. All relevant data are within the paper and its S3 Table.

## Results

### Development of specific serodiagnostic assays

**Fig 2:**
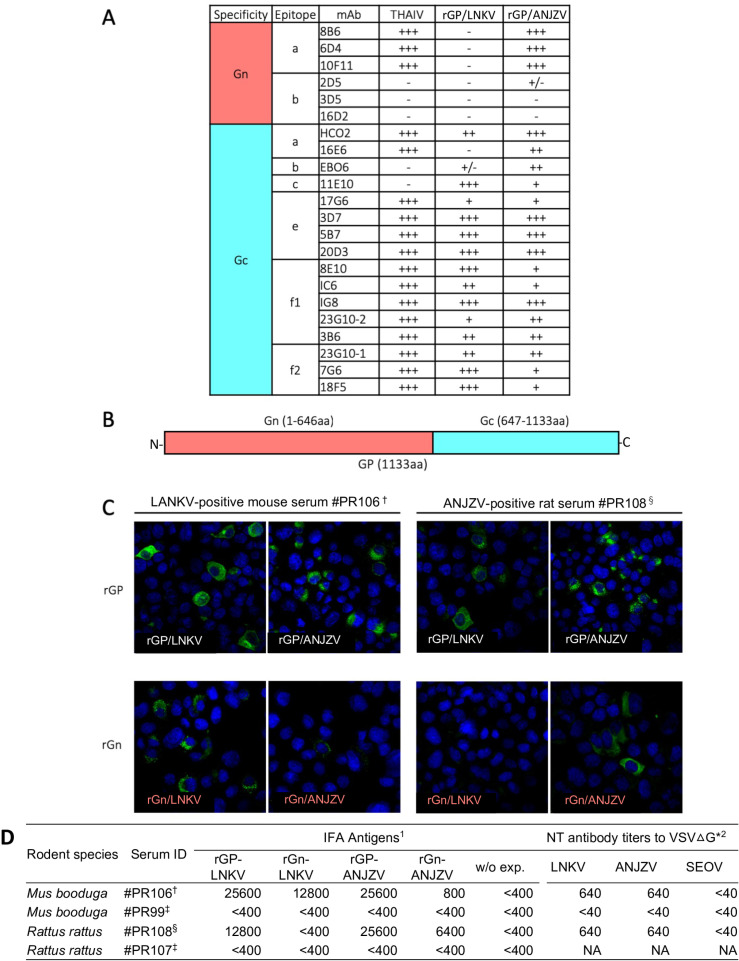
Establishment of the serotyping method for LNKV and ANJZV infection based on the antigenic analysis of rGPs. A: Antigenic profiling of rGPs of LNKV and ANJZV. The epitopes are mentioned as described by Arikawa et. al., [29]. B: Schematic diagram representing the arrangement of Gn and Gc of two virus GPs used in this study. C: IFA profiles of sera from host rodents at the serum dilution 1:1600. † Anti-LNKV serum from genome-positive and IgG-positive *Mus booduga* #PR106. § Anti-ANJZV serum from genome-positive and IgG-positive *Rattus rattus* #PR108. D: IFA titers and cross-neutralization titers of rodent sera used in panel C. As antibody negatives, sera from IgG-negative and genome-negative rodents, *Mus booduga* #PR99 ‡ and *Rattus rattus* #PR107 ‡ were used. ^1^Recombinant IFA antigens were transiently expressed in BHK/T7-9 cells and used for the endpoint titer determination. 2 Methods related to the alternative neutralization assay are shown in the S1 Text.

To understand the antigenic characteristics of the rGP antigens of LNKV and ANJZV viruses, reactivities of monoclonal antibodies directed to HTNV GP were examined. Interestingly, a unique antigenic pattern was identified in the Gn region of LNKV, which was absent in the prototype THAIV and ANJZV (Fig 2A and 2B). Based on these observations, the Gn regions of LNKV and ANJZV were expressed in BHK/T7-9 cells and served as serotyping antigens. Finally, we confirmed the reactivity of the screening and serotyping antigens by allowing them to react with the sera of rodents naturally infected with LNKV and ANJZV. As shown in Fig 2C, the IFA signal against heterologous rGn antigens decreased when they reacted with virus-infected rodent sera. The heterologous endpoint of rodent serum was also reduced (Fig 2D). Although we established an alternative neutralization assay by using a pseudotype virus system, neutralization titers were not useful to differentiate LNKV and ANJZV infection.

### Hantavirus infection in febrile patients from CKDu endemic regions

We collected 181 human serum samples from patients with acute fever in two CKDu-endemic regions (Polonnaruwa- 94; Girandurukotte- 87). Clinical characteristics of febrile patients are shown in S2 Table. As shown in Table 1, two (1.0%) and 61 (33.7%) samples were positive for anti-hantavirus IgM and IgG antibodies, respectively. Of 61 IgG positives, 52 were further serotyped, identifying 30 samples as LNKV infection, and the remaining 22 were inconclusive, suggesting that LNKV might be the primary source of infection in these areas.

**Table 1:** Hantavirus antibody positivity in febrile patients.

Year	Area	Collected No.	IgM ELISA positive	THAIV IFA positive	rGP IFA positive	rGn IFA serotyping		
						LNKV	ANJZV	Inc.
2022^*^	Polonnaruwa	94	2	36	30	15	0	15
	Girandurukotte	87	0	25	22	15	0	7
	Total	181	2	61	52	30	0	22
2016^†^	Unspecified	76	5	10	6	3	0	3

Inc.: Inconclusive,

* Sera from CKDu endemic regions used for prospective analyses,

† Sera from hospitalized acute patients used for retrospective analyses.

**Fig 3:**
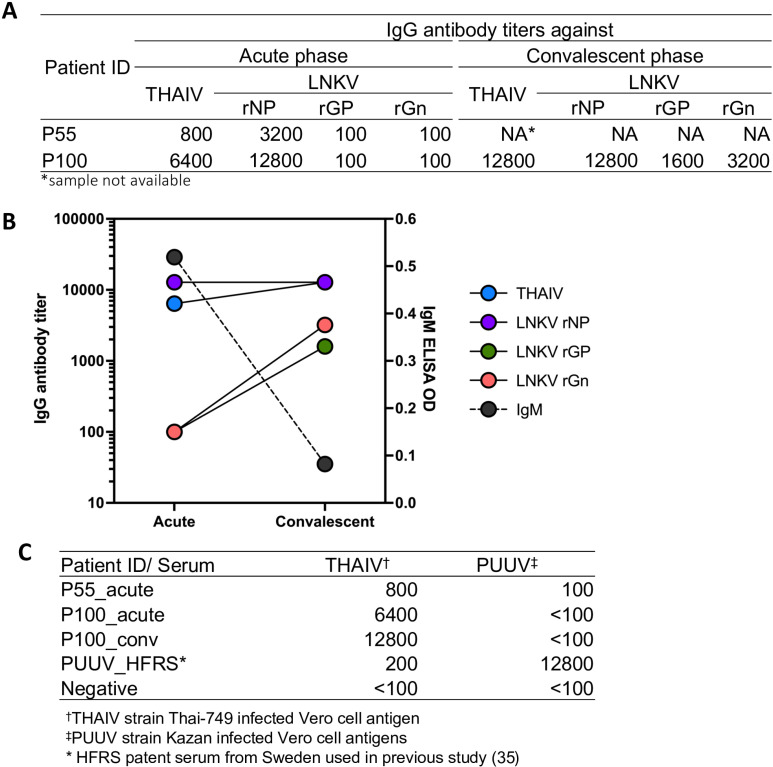
Antibody titers in LNKV genome-positive febrile patient sera. (A) Anti-LNKV antibody titers of P55 acute-serum and P100 acute and convalescent sera. (B) Serum antibody fluctuation graph of P100 at acute and convalescent phase sera. (C) Cross-reactivities of P55 and P100 to PUUV antigen. [35].

Hantavirus genome screening was then performed in all 61 IgG-seropositives including two samples positive for both IgG and IgM. Two (3.2%) genome-positive samples were identified from two patients who were positive for IgM and IgG (patient IDs: Pol/2022/055 [P55] and Pol/2022/100 [P100]). P55 had two days of fever at the time of sample collection, whereas P100 was on fever day 3. Both patients were male (P55: 29 years; P100: 43 years) with no renal dysfunction and presented with acute fever, headache, and myalgia. In addition, P100 experienced prostration. After three months, the P100 patient visited the hospital and was permitted to examine his serum as a convalescent-phase specimen. Details of the serological assays are shown in Fig 3. In the acute phase, the two patients already had IgG antibodies but lacked anti-GP antibodies. After three months of onset, anti-GP antibodies were induced and able to be serotyped. As shown in Fig 3A and 3B, P100 showed the LNKV infection pattern in the rGn-based serotyping assay.

### Phylogenetic analysis of sequences obtained from febrile patients

**Fig 4:**
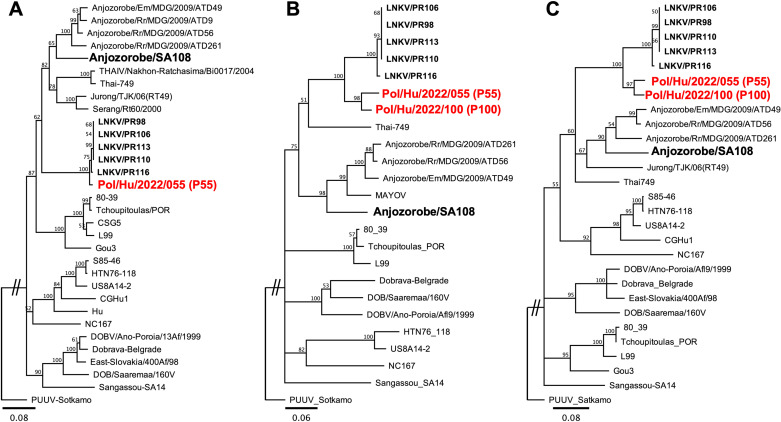
Neighbor-Joining phylogenetic trees of partial nucleotide sequences. (A) L-segment (347 bp: nt 2966-3312) (B) M-segment (218 bp: nt 2055-2272) and (C) S-segment (568 bp: nt 27-594), of newfound human hantaviruses and representative *Muridae*-borne hantaviruses. Representative *Muridae*-borne hantavirus sequences retrieved from databases were used to compare with newfound virus sequences (in boldface red) in this study. Previously identified *Mus*-borne LNKV sequences and rat-borne ANJZV sequences from Sri Lanka are in boldface. The scale bar indicates the sequence divergence values. The numbers above the nodes indicate the percentage consensus support values. The accession numbers of the sequences retrieved from the databases are mentioned in the legend of S1 Fig in the Supporting file.

Phylogenetic analysis revealed that the two L-genome fragments obtained from patient sera were similar to the LNKV sequences identified from Sri Lankan little Indian field mouse strains, as previously reported [28] (Fig 4A). PCR amplification of the M-segment sequences yielded a 271-bp fragment from both patient samples. However, only one patient sample (P55) yielded a 620-bp S-genome fragment that could be sequenced. We observed that the viral RNA load in the acute-phase P100 serum was lower than that in P55 since the amplification of genome fragments in P100 was weaker, failing to amplify the S segment even after many attempts. The M- and S-genome fragment sequences were also grouped with the LNKV lineage detected in little Indian field mouse, with some nucleotide differences (Fig 4B and 4C). The deduced partial amino acid sequences showed the same results (S1 Fig). These results suggest that both patients were infected with LNKV. The partial hantavirus genome sequences determined in this study have been deposited in a public database with the GenBank accession numbers LC764432 – LC764436.

### Retrospective analysis of patients with fever of unknown origin

Serum samples from patients with acute febrile illness of unknown origin received by the Department of Microbiology, Peradeniya University, from different hospitals in 2016 were first analyzed for anti-hantavirus antibodies and the hantavirus genome. Of the 76 serum samples, five (6.6%) and ten (13.2%) tested positive for IgM and IgG antibodies, respectively (Table 1). Viral RNA was not detected in any of these samples. Of ten IgG-positives, six sera having anti-GP antibodies were further serotyped to identify three samples as LNKV infection, with the others being inconclusive, suggesting that LNKV may cause acute illness. Details of the serological assays are shown in Table 2. The patients’ residences included not only CKDu-endemic areas but also non-endemic areas such as Kandy.

**Table 2:** Details of retrospective serological test results for patient sera with acute fever of unknown origin.

Patient ID^*^	Age	Gender	Antibody screening			Antibody titers to		Serotype/ result	Patient living area
			IgM ELISA	THAIV IFA	rGP IFA	LNKV rGn	ANJZV rGn		
69H	41	M	+	+	+	6400	800	LNKV	Kandy
118H	25	M	+	+/-	–	ND	ND	ND	Hatton
144H^*^	65	M	–	+	–	ND	ND	ND	Not recorded
164H	29	M	–	+	+	12800	3200	LNKV	Polonnaruwa
169H	46	M	–	+	+	12800	6400	Inc.	Kandy
173H	39	M	+	+	+	6400	400	LNKV	Polonnaruwa
174H^*^	46	M	+	+	+	400	200	Inc.	Kandy
175H^*^	43	M	+	+	+	1600	800	Inc.	Polonnaruwa
179H	70	M	–	+	–	ND	ND	ND	Kandy
OPD1	52	F	–	+	–	ND	ND	ND	Girandurukotte

* Cases treated in intensive care units, ND: Not determined, Inc.: Inconclusive.

### Retrospective serotyping of sera from patients with CKDu and healthy controls

We employed rGP-based IFA screening to select human sera from CKDu-endemic regions of Sri Lanka for serotyping. Of the 373 serum samples, 336 contained anti-GP antibodies and were thus selected for serotyping (Table 3, S2 Fig, S3 Table). By analyzing the ratio values between the EPs, we found that among the patient group, 184/210 (87.6%) and 2/210 (1.0%) were positive for LNKV and ANJZV, respectively. In the healthy control group, 110/126 (87.3%) individuals were tested positive for LNKV; no ANJZV-positive sera were identified. LNKV infected a significant proportion of patients with CKDu and healthy controls, although the difference in both proportions was not statistically significant (difference: 0.3%, 95% CI: -7.0–7.6%, p > 0.05).

**Table 3:** Details of the sample collections, geographical locations, and serological test results of CKDu patient and healthy control serum samples.

Condition	Year	Area	Collected No.	THAIV IFA positive	rGP IFA positive	rGn IFA serotyping^†^		
						LNKV	ANJZV	Inconclusive
CKDu	2010	Girandurukotte	151^*^	63 (42.0%)	62	53 (35.1%)	0	9 (6.0%)
		Medawachchiya	160^*^	65 (40.6%)	64	55 (34.4%)	0	9 (5.6%)
	2015	Girandurukotte	132	62 (49.2%)	51	47 (35.6%)	0	4 (3.0%)
	2017	Girandurukotte	104	52 (50.0%)	33	29 (27.9%)	2 (1.9%)	2 (1.9%)
CKDu total	··	··	547	242 (44.2%)	210	184 (33.6%)	2 (0.4%)	24 (4.4%)
Healthy	2010	Girandurukotte	150^*^	28 (18.7%)	28	26 (17.3%)	0	2 (1.3%)
		Medawachchiya	156^*^	24 (15.4%)	24	19 (12.2%)	0	5 (3.2%)
	2017	Girandurukotte	242	42 (17.4%)	42	38 (15.7%)	0	4 (1.6%)
	2019	Welikanda	59	12 (20.3%)	8	8 (13.6%)	0	0
	2019	Wilgamuwa	143^*^	25 (17.5%)	24	19 (13.3%)	0	5 (3.5%)
Healthy total	··	··	750	131 (17.4%)	126	110 (14.7%)	0	16 (2.1%)
Total	..	..	1297	373	336	294 (22.7%)	2 (0.2%)	40 (3.1%)

* The samples were collected from male subjects only due to the inclusion criteria of the corresponding studies.

† Percentages are given in relation to the initial collected number of samples of each group.

## Discussion

We previously reported the identification of many hantavirus antibody-positive individuals from CKDu-endemic areas of Sri Lanka [16,18]. While studies have shown a statistically significant association between orthohantavirus antibody positivity and CKDu in Sri Lanka [18,19,36], the fundamental nature of this association has not been fully elucidated. Recently, we also reported the genome information of two distinct hantaviruses from rodent populations in Sri Lanka [28]. In this study, we attempted to identify the hantaviruses that infect humans in Sri Lanka and their pathogenicity in humans.

In our results, we could only determine the sequences of short LNKV genome fragments from two febrile patients, highlighting the challenges associated with detecting the LNKV genome in humans. Specifically, the short viremic phase with nonspecific and/or mild symptoms during the acute phase of LNKV infection makes detecting the virus in infected individuals difficult. Furthermore, it is important to note that people in these areas do not usually visit hospitals when they experience mild fevers. Hence, it is challenging to estimate the frequency of LNKV infection in these areas based solely on the results of this study. It is also worth noting that the two LNKV genome-positive patients were found under unusual circumstances during the COVID-19 pandemic when people with fever were required to seek medical attention immediately. Fifty-five febrile patient sera from Polonnaruwa that were collected in February 2022 to use in this study were examined by a rapid COVID-19 diagnostic kit; however, all were tested negative. Further studies and surveillance are required to estimate the prevalence of LNKV infection.

As described above, detecting viral genomes from patients, and diagnosing them using molecular tools based on sequence similarity is the most reliable method to understand the prevalence of the viruses. However, because the viremic period of the patients appeared to be very short, it is also necessary to rely on serological methods to identify the infected virus in patients who have recovered or have late-phase infection. Since the gold standard assay for serotyping hantavirus infection is a neutralization test, as an initial attempt, we developed an alternative neutralization test based on a pseudotyped VSV system. Although high titers of neutralizing antibodies were detected in virus-infected animals, an equal degree of cross-neutralization was observed. Therefore, it was impossible to differentiate between LNKV and ANJZV infections using neutralization assays. Next, we used the unique antigenic region Gn as a novel antigen for the IFA assay to distinguish between the two viral infections. The corresponding viral infections were successfully distinguished by comparing the antibody titers against the LNKV and ANJZV Gn antigens.

Various serological tests were employed to examine the sera of patients with fever of unknown etiology collected in 2016. These serum samples were examined for IgM antibodies against HTNV and IgG antibodies against THAIV antigens before identifying Sri Lankan hantaviruses. After the initial analyses, these sera were stored at -20°C until this study, which may have caused the loss of the viral genome. In contrast, anti-GP antibodies were successfully detected, and three serum samples were serotyped as LNKV. One of the patients (ID: 174H), whose serum sample was received from Kandy General Hospital, Central Province, was admitted to the hospital 3 days after the onset of symptoms such as fever, headache, myalgia, arthralgia, and diarrhea. By day 4, the patient had developed acute kidney injury, high serum creatinine levels, pulmonary edema, and bleeding, requiring ventilatory support in the intensive care unit. Blood samples from this patient obtained on day 18 from symptom onset harbored both IgM and IgG antibodies [13]. Because the induction of anti-GP antibodies was insufficient, we failed to serotype 174H. Patients 144H and 175H also required treatments in the intensive care units, but we failed to serotype these three severe cases. Although the anti-GP-based strategy is inapplicable during the acute phase, genome detection by PCR is applicable. Sharing such findings with the doctors in clinical wards is essential to diagnose LNKV infection.

As shown in previous studies, we identified many seropositive individuals from several CKDu hotspot regions in Sri Lanka [19]. More than 87% of GP antibody-positive samples were serotyped as LNKV infections. The serotyping strategy developed in this study is useful for seroepidemiological studies to detect past infections.

We previously reported a high seroprevalence of anti-hantavirus antibodies in *R. rattus* populations in the Girandurukotte region [20]. Epizootiological analysis suggests that many rats may have been infected with LNKV via spillover, and subsequently, their bodies eliminated the virus. LNKV may be excreted from mice into the environment, providing many opportunities for infection in rats and humans. However, the distribution of ANJZV-related virus in Sri Lanka is still unknown. Therefore, further field studies are needed to investigate viruses and their hosts. The possibility of having other circulating hantaviruses that are yet to be discovered must also be considered.

*M. booduga*, commonly known as the little Indian field mouse, is mostly found in agricultural fields, shrublands, and forests [37]. This information is important when considering public health measures for controlling this zoonotic LNKV. However, to truly clarify the hazardous risk to human health posed by the little Indian field mouse and LNKV, analyses of more human cases and rodents are required.

In conclusion, the findings of this study reveal the genomic basis and serotypes of hantaviruses that infect humans in Sri Lanka. It appears that the disease caused by LNKV infection can vary from a mild febrile illness, which is predominant, to severe illnesses demanding supportive care in certain individuals. Unlike other THAIV-like viruses, LNKV can cause large-scale human infections, warranting more detailed epidemiological studies to understand the island-wide distribution of LNKV and its reservoir hosts. Furthermore, the isolation of this unique *Mus*-borne virus will allow for more in-depth research on its pathogenesis in humans.

## Supporting information

S1 Fig Neighbor-Joining phylogenetic trees of deduced partial amino-acid sequences of (A) L-protein, (B) GP, and (C) N protein of newfound human hantaviruses and representative *Muridae*-borne hantaviruses. Representative *Muridae*-borne hantavirus genome sequences retrieved from databases were used to deduce the amino-acid sequences to compare with those of newfound virus sequences (in boldface red) in this study. Previously identified *Mus*-borne LNKV sequences and rat-borne ANJZV sequences from Sri Lanka are in boldface. The scale bar indicates the sequence divergence values. The numbers above the nodes indicate the percentage consensus support values. Hantaan (HTNV): HTN76–118 (L: X55901, M: M14627, S: M14626), US8A14-2 (L: KU207200, M: KU207204, S: KU207208), S85-46 (M: AF288658, S: AF288659), CGHu1 (M: EU092222, S: EU092218), and Hu (S: AB027111); Dabieshan: NC167 (L: DQ989237, M: AB027115, S: AB027523); Seoul (SEOV): 80–39 (L: X56492, M: S47716, S: AY273791), Tchoupitoulas-POR (L: KU204958, M: KU204959, S: KU204960), L99 (L: AF288297, M: AF035833, S: AF288299), Gou3 (M: AF145977, S: AF184988), and CSG5 (S: AB618112); Dobrava (DOBV): DOBV/Ano-Poroia/Afl9/1999 (L: AJ410617, M: AJ410616, S: AJ410615), Dobrava-Belgrade (L: JQ026206, M: L33685, S: L41916), DOB/Saaremaa/160V (L: AJ410618, M: AJ009774, S: AJ009773), and East Slovakia/400Af/98 (S: AY168576); Sangassou: SA14 (L: JQ082302, M: JQ082301, S: JQ082303); Thailand (THAIV): Thai-749 (L: LC553715, M: L08756, S: AB186420), Nakhon Ratchasima/Bi0017/2004 (S: AM397664), ANJZV strain Anjozorobe/Em/MDG/2009/ATD49 (L: KC490922, M: KC490919, S: KC490918), ANJZV strain Anjozorobe/Rr/MDG/2009/ATD56 (L: KC490923, M: KC490921, S: KC490916), ANJZV strain Anjozorobe/Rr/MDG/2009/ATD261 (L: KC490924, M: KC490920, S: KC490914), ANJZV strain Anjozorobe/Rr/MDG/2009/ATD9 (S: KC490915), ANJZV strain Anjozorobe/2019/PR108 (L: LC553724, M: LC553723, S: LC553722), Jurong strain TJK/06/RT49 (M: GQ274938, S: GQ274940), Serang strain Serang/Rt60/2000 (S: AM998808), Mayotte strain MAYOV (L: KU587796); Lanka (LNKV): Lanka/2018/PR98 (L: LC553718, M: LC553717, S: LC553716), Lanka/2018/PR106 (L: LC553721, M: LC553720, S: LC553719), Lanka/2018/PR110 (L: LC553727, M: LC553726, S: LC553725), Lanka/2018/PR113 (L: LC553730, M: LC553729, S: LC553728), Lanka/2018/PR116 (L: LC553733, M: LC553732, S: LC553731); and Puumala (PUUV):Sotkamo (L: Z66548, M: X61034, S:X61035).
(TIF)

S2 Fig Comparison of antibody titers to LNKV and ANJZV in CKDu patients and healthy individuals from CKDu endemic areas in Sri Lanka. IFA antibody endpoint titers were determined and plotted. The area of the points reflects the number of samples. Samples showing four times or higher titer to LNKV antigen than to ANJZV antigen are displayed in red as suspected LNKV infection. Conversely, samples showing four times or higher titer against the ANJZV antigen than the LNKV antigen are shown in blue as suspected ANJZV infection. Samples with an antibody titer difference of less than 2 times are shown in white areas as “inconclusive”.
(TIF)

S1 Table Details of the primers used for the LNKV genome amplification from febrile serum samples and cloning of NP, GP, and Gn of LNKV and ANJZV. (DOCX)

S2 Table Clinical characteristics of febrile patients from Polonnaruwa (PN) and Girandrukotte (GK). (DOCX)

S3 Table The serotyping IFA endpoint titer data. The counts of CKDu patient and healthy serum sample end-point titers against ANJZV and LNKV rGn antigens.
(DOCX)

S1 Text Supporting methods. (DOCX)
