# Supplementary figures and images for: Lanka virus, a Mus booduga-borne orthohantavirus infection-associated febrile illness in Sri Lanka
Source: PLoS Negl Trop Dis. 2025 Jun 11;19(6):e0013169. doi: 10.1371/journal.pntd.0013169 (PMC12193775; doi:10.1371/journal.pntd.0013169)

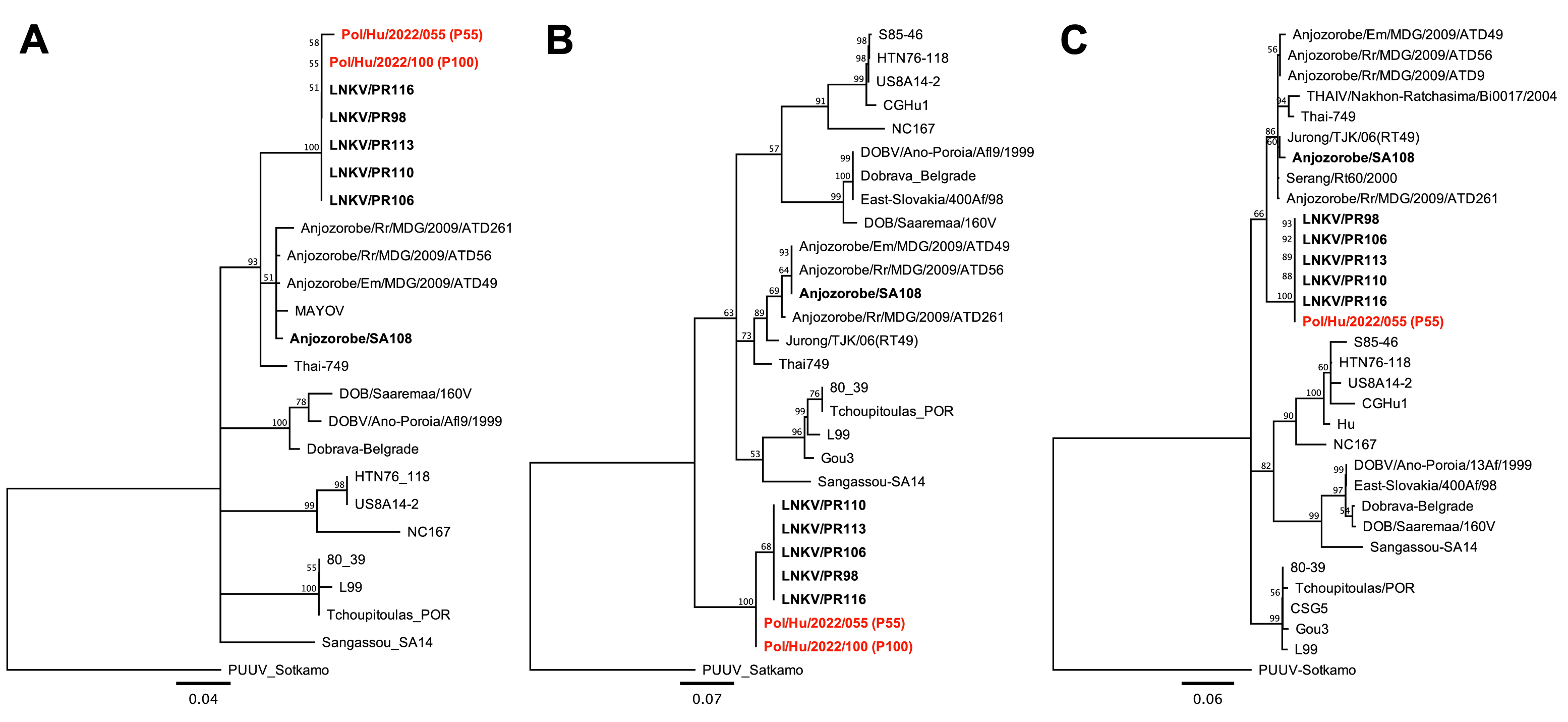

Supplement: S1 Fig — Representative Muridae-borne hantavirus genome sequences retrieved from databases were used to deduce the amino-acid sequences to compare with those of newfound virus sequences (in boldface red) in this study. Previously identified Mus-borne LNKV sequences and rat-borne ANJZV sequences from Sri Lanka are in boldface. The scale bar indicates the sequence divergence values. The numbers above the nodes indicate the percentage consensus support values. Hantaan (HTNV): HTN76–118 (L: X55901, M: M14627, S: M14626), US8A14-2 (L: KU207200, M: KU207204, S: KU207208), S85-46 (M: AF288658, S: AF288659), CGHu1 (M: EU092222, S: EU092218), and Hu (S: AB027111); Dabieshan: NC167 (L: DQ989237, M: AB027115, S: AB027523); Seoul (SEOV): 80–39 (L: X56492, M: S47716, S: AY273791), Tchoupitoulas-POR (L: KU204958, M: KU204959, S: KU204960), L99 (L: AF288297, M: AF035833, S: AF288299), Gou3 (M: AF145977, S: AF184988), and CSG5 (S: AB618112); Dobrava (DOBV): DOBV/Ano-Poroia/Afl9/1999 (L: AJ410617, M: AJ410616, S: AJ410615), Dobrava-Belgrade (L: JQ026206, M: L33685, S: L41916), DOB/Saaremaa/160V (L: AJ410618, M: AJ009774, S: AJ009773), and East Slovakia/400Af/98 (S: AY168576); Sangassou: SA14 (L: JQ082302, M: JQ082301, S: JQ082303); Thailand (THAIV): Thai-749 (L: LC553715, M: L08756, S: AB186420), Nakhon Ratchasima/Bi0017/2004 (S: AM397664), ANJZV strain Anjozorobe/Em/MDG/2009/ATD49 (L: KC490922, M: KC490919, S: KC490918), ANJZV strain Anjozorobe/Rr/MDG/2009/ATD56 (L: KC490923, M: KC490921, S: KC490916), ANJZV strain Anjozorobe/Rr/MDG/2009/ATD261 (L: KC490924, M: KC490920, S: KC490914), ANJZV strain Anjozorobe/Rr/MDG/2009/ATD9 (S: KC490915), ANJZV strain Anjozorobe/2019/PR108 (L: LC553724, M: LC553723, S: LC553722), Jurong strain TJK/06/RT49 (M: GQ274938, S: GQ274940), Serang strain Serang/Rt60/2000 (S: AM998808), Mayotte strain MAYOV (L: KU587796); Lanka (LNKV): Lanka/2018/PR98 (L: LC553718, M: LC553717, S: LC553716), Lanka/2018/PR106 (L: LC553721, M: LC553720, S: LC553719), Lanka/2018/PR110 [file pntd.0013169.s001.tif]

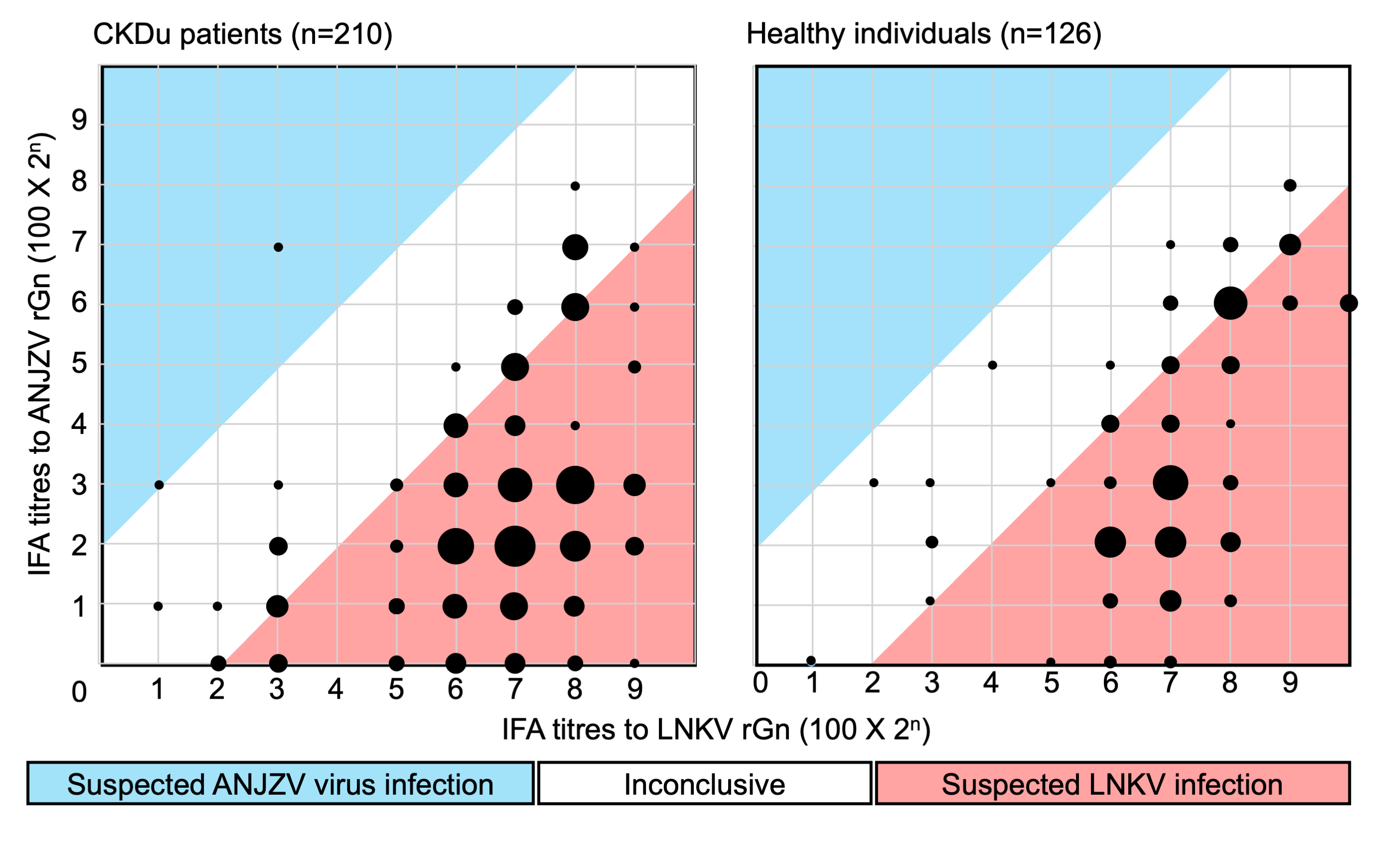

Supplement: S2 Fig — IFA antibody endpoint titers were determined and plotted. The area of the points reflects the number of samples. Samples showing four times or higher titer to LNKV antigen than to ANJZV antigen are displayed in red as suspected LNKV infection. Conversely, samples showing four times or higher titer against the ANJZV antigen than the LNKV antigen are shown in blue as suspected ANJZV infection. Samples with an antibody titer difference of less than 2 times are shown in white areas as “inconclusive”. (TIF) [file pntd.0013169.s002.tif]
