# Supplementary material for: Lanka virus, a Mus booduga-borne orthohantavirus infection-associated febrile illness in Sri Lanka
Source: PLoS Negl Trop Dis. 2025 Jun 11;19(6):e0013169. doi: 10.1371/journal.pntd.0013169 (PMC12193775; doi:10.1371/journal.pntd.0013169)
Supplement: S1 Table — (DOCX) [file pntd.0013169.s003.docx]

**Supporting Information File**

**Supporting Methods**

Methods for alternative neutralization assay

**Preparation of pseudotype viruses.**

Pseudotype viruses were prepared as previously described (36). Briefly, for the preparation of pseudotype viruses bearing LNKV and ANJZV GPs, a recombinant vesicular stomatitis virus (VSV) derived from a full-length cDNA clone of the VSV genome (Indiana serotype) in which the coding region of the G protein was replaced by the coding region of the GFP gene and the G protein was expressed in trans designated as VSV△G*G was used as the stock virus. At 36 hours after transfection of 293T cells with pCLNK-M and pCANJZ-M, the cells were infected with VSV△G*G at a multiplicity of infection of 1 for 1 hour at room temperature. The 293T cell monolayer was then washed with 1% heat-inactivated FCS-PBS three times, and the culture medium was added. After 48 hours of incubation at 37°C in a CO_2_ incubator, the culture supernatant was clarified by low-speed centrifugation and stored at -80°C. The pseudotype viruses bearing LNKV and ANJZV GPs were designated as VSV△G*LNKV and VSV△G*ANJZV, respectively.

**Titration of pseudotype viruses.**

For pseudotype virus titration, Vero E6 cell monolayers grown on 96-well plates were infected with 50 µL of serially diluted virus stock. After a 1-hour adsorption period, the inoculum was removed, fresh culture medium was added, and the cells were incubated at 37°C in a CO_2_ incubator. At 16 hours post-infection, the cells were fixed with 2% paraformaldehyde in PBS for 10 min at room temperature, washed with distilled water, and air dried. GFP-expressing cells were counted under a fluorescence microscope. Since pseudotype VSVs are unable to produce infectious progeny virus, the numbers of GFP-positive cells were regarded as infectious units (IU).

**Neutralization of VSV pseudotypes.**

A total of 30 µL of medium containing 100 IU of prepared VSV pseudotypes was incubated with an equal volume of serially diluted rodent sera for 1 h at 37°C. Then, 50 µL of the mixture was inoculated onto Vero E6 cell monolayers in 96-well tissue culture plates. After adsorption for 1 hour, the mixture was replaced with Eagle minimal essential medium. After a 20-hour incubation period, the cells were fixed with 2% paraformaldehyde for 10 min, washed with distilled water, and air dried. Cells infected with VSV pseudotypes were examined and counted based on GFP expression under a fluorescence microscope. 80% reduction of GFP-expressing cells was considered for the neutralizing titer determination.

| Purpose | Primer ID | Sequence (5'-3') |
| --- | --- | --- |
| LNKV S- segment amplification | Muridae_hanta S1F | TAGTAGTAGICTICSTRAARAGCTAC |
|  | Lanka S620R | TCCTCAGCTTTCATGCTTGACTGGGC |
|  | Lanka S707R | CTCATCACTGGACTGACCATATTCCTAGC |
| LNKV M- segment amplification | THAIV-like M2003F | CCACTCTGGACWGAYAATGCWCATGG |
|  | THAIV-like M2030F | ATTGGITCWGTYCCIATGCAYACTG |
|  | THAIV-like M2301R | TCAAAGTGRCACTTGGCTGTATGCCAAGG |
| LNKV S cloning | Lanka_S_F1_EcoR1 | CCCGAATTCATGGCAAGTATGGAGGAGTTA |
|  | Lanka_N_Sal1 | TTTGTCGACTTAAAGCTTTAAGG |
| ANJZV S cloning | PR108 NF SacI | TTGGCAAAGAATTCGAGCTCATGGCAACTATGGAAGAGTTACAAAG |
|  | PR108 NR XhoI | GATCTGCTAGCTCGAGTTAGAGCTTCAGGGGCTCTTGGTTAG |
| LNKV M and Gn cloning | Lanka_M47F_Cla1 | AACATCGATATGTGGGGTTTACTAGCTTGGCTGC |
|  | Lanka_M1965R_Xho1 | GGACTCGAGTTATGCACTAGCTGCCCATAA GACTG |
|  | Lanka_M3448R_NheI | GCTGCTAGCTTATGACTTCTTATGCTTACGCACAGGG |
| ANJZV M and Gn cloning | PR108_M47F_Sac1 | TCATTTTGGCAAAGAATTCGAGCTCATGTGGGGTTTACTAGCTATTGCTGTTTTG |
|  | PR108_M1965R_Xho1 | GGACTCGAGTTATGCACTTGCAGCCCACAT TATAGAC |
|  | PR108_M3448R_Xho1 | AAAAAGATCTGCTAGCTCGAGTTATGATTTCTTGTGCTTGCGAAC |

**S1 Table**. Details of the primers used for the LNKV genome amplification from febrile serum samples and cloning of NP, GP, and Gn of LNKV and ANJZV.

| Variable | Total no. | | No. IgG positive patients | | No. IgM positive patients | |
| --- | --- | --- | --- | --- | --- | --- |
|  | PN (n=94) | GK (n=87) | PN (n=36) | GK (n=25) | PN (n=2) | GK (n=0) |
| Sex | | | | | | |
| Female | 32 | 32 | 8 | 7 | 0 | 0 |
| Male | 62 | 55 | 28 | 18 | 2 | 0 |
| Patient information | | | | | | |
| Days of fever | 3.28 | 2.82 | 3.28 | 2.76 | 2.5 | 0 |
| Age | 41.1 | 44.9 | 45.6 | 47.6 | 36.0 | 0 |
| Occupation |  |  |  |  |  |  |
| Agriculture | 46 | 55 | 18 | 17 | 1 | 0 |
| Non-agriculture | 48 | 32 | 18 | 8 | 1 | 0 |
| Clinical features | | | | | | |
| Headache | 76 | 73 | 30 | 20 | 2 | 0 |
| Myalgia | 66 | 70 | 26 | 21 | 2 | 0 |
| Cough | 33 | 21 | 12 | 6 | 0 | 0 |
| Prostration | 10 | 20 | 5 | 7 | 1 | 0 |
| Breathlessness | 10 | 13 | 5 | 4 | 0 | 0 |
| Anuria/oliguria | 2 | 0 | 1 | 0 | 0 | 0 |
| Conjunctival suffusion | 8 | 0 | 4 | 0 | 0 | 0 |
| Jaundice | 8 | 3 | 3 | 0 | 0 | 0 |
| Skin rash | 1 | 0 | 0 | 1 | 0 | 0 |
| Hemoptysis | 0 | 1 | 0 | 1 | 0 | 0 |
| Admitted to the hospital | | | | | | |
| Yes | 31 | 5 | 12 | 2 | 1 | 0 |
| No | 63 | 82 | 24 | 23 | 1 | 0 |

**S2 Table**. Clinical characteristics of febrile patients from Polonnaruwa (PN) and Girandrukotte (GK)

| Endpoint titer  (2^n × 100) | | Positive count | |
| --- | --- | --- | --- |
| ANJZV-rGn | LNKV-rGn | CKDu | Healthy |
| 0 | 1 | 0 | 1 |
| 0 | 2 | 3 | 0 |
| 0 | 3 | 4 | 1 |
| 0 | 4 | 0 | 0 |
| 0 | 5 | 3 | 1 |
| 0 | 6 | 5 | 2 |
| 0 | 7 | 5 | 3 |
| 0 | 8 | 3 | 0 |
| 0 | 9 | 1 | 0 |
| 0 | 10 | 0 | 0 |
| 1 | 1 | 1 | 0 |
| 1 | 2 | 1 | 0 |
| 1 | 3 | 6 | 1 |
| 1 | 4 | 0 | 0 |
| 1 | 5 | 3 | 0 |
| 1 | 6 | 7 | 4 |
| 1 | 7 | 9 | 6 |
| 1 | 8 | 5 | 2 |
| 1 | 9 | 0 | 0 |
| 1 | 10 | 0 | 0 |
| 2 | 1 | 0 | 0 |
| 2 | 2 | 0 | 0 |
| 2 | 3 | 4 | 3 |
| 2 | 4 | 0 | 0 |
| 2 | 5 | 2 | 0 |
| 2 | 6 | 15 | 12 |
| 2 | 7 | 19 | 12 |
| 2 | 8 | 10 | 5 |
| 2 | 9 | 4 | 0 |
| 2 | 10 | 0 | 0 |
| 3 | 1 | 1 | 0 |
| 3 | 2 | 0 | 1 |
| 3 | 3 | 4 | 1 |
| 3 | 4 | 0 | 0 |
| 3 | 5 | 2 | 1 |
| 3 | 6 | 7 | 4 |
| 3 | 7 | 14 | 15 |
| 3 | 8 | 17 | 3 |
| 3 | 9 | 6 | 0 |
| 3 | 10 | 0 | 0 |
| 4 | 1 | 0 | 0 |
| 4 | 2 | 0 | 0 |
| 4 | 3 | 0 | 0 |
| 4 | 4 | 0 | 0 |
| 4 | 5 | 0 | 1 |
| 4 | 6 | 7 | 5 |
| 4 | 7 | 5 | 5 |
| 4 | 8 | 1 | 1 |
| 4 | 9 | 0 | 0 |
| 4 | 10 | 0 | 0 |
| 5 | 1 | 0 | 0 |
| 5 | 2 | 0 | 0 |
| 5 | 3 | 0 | 1 |
| 5 | 4 | 0 | 1 |
| 5 | 5 | 0 | 0 |
| 5 | 6 | 1 | 1 |
| 5 | 7 | 9 | 4 |
| 5 | 8 | 0 | 4 |
| 5 | 9 | 2 | 0 |
| 5 | 10 | 0 | 0 |
| 6 | 1 | 0 | 0 |
| 6 | 2 | 0 | 0 |
| 6 | 3 | 0 | 0 |
| 6 | 4 | 0 | 0 |
| 6 | 5 | 0 | 0 |
| 6 | 6 | 0 | 0 |
| 6 | 7 | 3 | 3 |
| 6 | 8 | 9 | 14 |
| 6 | 9 | 1 | 3 |
| 6 | 10 | 0 | 4 |
| 7 | 1 | 0 | 0 |
| 7 | 2 | 0 | 0 |
| 7 | 3 | 1 | 0 |
| 7 | 4 | 0 | 0 |
| 7 | 5 | 0 | 0 |
| 7 | 6 | 0 | 0 |
| 7 | 7 | 0 | 1 |
| 7 | 8 | 8 | 3 |
| 7 | 9 | 1 | 6 |
| 7 | 10 | 0 | 0 |
| 8 | 1 | 0 | 0 |
| 8 | 2 | 0 | 0 |
| 8 | 3 | 0 | 0 |
| 8 | 4 | 0 | 0 |
| 8 | 5 | 0 | 0 |
| 8 | 6 | 0 | 0 |
| 8 | 7 | 0 | 0 |
| 8 | 8 | 1 | 0 |
| 8 | 9 | 0 | 3 |
| 8 | 10 | 0 | 0 |
| 9 | 1 | 0 | 0 |
| 9 | 2 | 0 | 0 |
| 9 | 3 | 0 | 0 |
| 9 | 4 | 0 | 0 |
| 9 | 5 | 0 | 0 |
| 9 | 6 | 0 | 0 |
| 9 | 7 | 0 | 0 |
| 9 | 8 | 0 | 0 |
| 9 | 9 | 0 | 0 |
| 9 | 10 | 0 | 0 |
| 10 | 1 | 0 | 0 |
| 10 | 2 | 0 | 0 |
| 10 | 3 | 0 | 0 |
| 10 | 4 | 0 | 0 |
| 10 | 5 | 0 | 0 |
| 10 | 6 | 0 | 0 |
| 10 | 7 | 0 | 0 |
| 10 | 8 | 0 | 0 |
| 10 | 9 | 0 | 0 |
| 10 | 10 | 0 | 0 |

**S3 Table.** The serotyping IFA endpoint titer data. The counts of CKDu patient and healthy serum sample end-point titers against ANJZV and LNKV rGn antigens.
