# Supplementary material for: Lanka virus, a Mus booduga-borne orthohantavirus infection-associated febrile illness in Sri Lanka
Source: PLoS Negl Trop Dis. 2025 Jun 11;19(6):e0013169. doi: 10.1371/journal.pntd.0013169 (PMC12193775; doi:10.1371/journal.pntd.0013169)
Supplement: S2 Table — (DOCX) [file pntd.0013169.s004.docx]

| Variable | Total no. | | No. IgG positive patients | | No. IgM positive patients | |
| --- | --- | --- | --- | --- | --- | --- |
|  | PN (n=94) | GK (n=87) | PN (n=36) | GK (n=25) | PN (n=2) | GK (n=0) |
| Sex | | | | | | |
| Female | 32 | 32 | 8 | 7 | 0 | 0 |
| Male | 62 | 55 | 28 | 18 | 2 | 0 |
| Patient information | | | | | | |
| Days of fever | 3.28 | 2.82 | 3.28 | 2.76 | 2.5 | 0 |
| Age | 41.1 | 44.9 | 45.6 | 47.6 | 36.0 | 0 |
| Occupation |  |  |  |  |  |  |
| Agriculture | 46 | 55 | 18 | 17 | 1 | 0 |
| Non-agriculture | 48 | 32 | 18 | 8 | 1 | 0 |
| Clinical features | | | | | | |
| Headache | 76 | 73 | 30 | 20 | 2 | 0 |
| Myalgia | 66 | 70 | 26 | 21 | 2 | 0 |
| Cough | 33 | 21 | 12 | 6 | 0 | 0 |
| Prostration | 10 | 20 | 5 | 7 | 1 | 0 |
| Breathlessness | 10 | 13 | 5 | 4 | 0 | 0 |
| Anuria/oliguria | 2 | 0 | 1 | 0 | 0 | 0 |
| Conjunctival suffusion | 8 | 0 | 4 | 0 | 0 | 0 |
| Jaundice | 8 | 3 | 3 | 0 | 0 | 0 |
| Skin rash | 1 | 0 | 0 | 1 | 0 | 0 |
| Hemoptysis | 0 | 1 | 0 | 1 | 0 | 0 |
| Admitted to the hospital | | | | | | |
| Yes | 31 | 5 | 12 | 2 | 1 | 0 |
| No | 63 | 82 | 24 | 23 | 1 | 0 |

**S2 Table**. Clinical characteristics of febrile patients from Polonnaruwa (PN) and Girandrukotte (GK)
