# Supplementary material for: Lanka virus, a Mus booduga-borne orthohantavirus infection-associated febrile illness in Sri Lanka
Source: PLoS Negl Trop Dis. 2025 Jun 11;19(6):e0013169. doi: 10.1371/journal.pntd.0013169 (PMC12193775; doi:10.1371/journal.pntd.0013169)
Supplement: S3 Table — The counts of CKDu patient and healthy serum sample end-point titers against ANJZV and LNKV rGn antigens. (DOCX) [file pntd.0013169.s005.docx]

| Endpoint titer  (2^n × 100) | | Positive count | |
| --- | --- | --- | --- |
| ANJZV-rGn | LNKV-rGn | CKDu | Healthy |
| 0 | 1 | 0 | 1 |
| 0 | 2 | 3 | 0 |
| 0 | 3 | 4 | 1 |
| 0 | 4 | 0 | 0 |
| 0 | 5 | 3 | 1 |
| 0 | 6 | 5 | 2 |
| 0 | 7 | 5 | 3 |
| 0 | 8 | 3 | 0 |
| 0 | 9 | 1 | 0 |
| 0 | 10 | 0 | 0 |
| 1 | 1 | 1 | 0 |
| 1 | 2 | 1 | 0 |
| 1 | 3 | 6 | 1 |
| 1 | 4 | 0 | 0 |
| 1 | 5 | 3 | 0 |
| 1 | 6 | 7 | 4 |
| 1 | 7 | 9 | 6 |
| 1 | 8 | 5 | 2 |
| 1 | 9 | 0 | 0 |
| 1 | 10 | 0 | 0 |
| 2 | 1 | 0 | 0 |
| 2 | 2 | 0 | 0 |
| 2 | 3 | 4 | 3 |
| 2 | 4 | 0 | 0 |
| 2 | 5 | 2 | 0 |
| 2 | 6 | 15 | 12 |
| 2 | 7 | 19 | 12 |
| 2 | 8 | 10 | 5 |
| 2 | 9 | 4 | 0 |
| 2 | 10 | 0 | 0 |
| 3 | 1 | 1 | 0 |
| 3 | 2 | 0 | 1 |
| 3 | 3 | 4 | 1 |
| 3 | 4 | 0 | 0 |
| 3 | 5 | 2 | 1 |
| 3 | 6 | 7 | 4 |
| 3 | 7 | 14 | 15 |
| 3 | 8 | 17 | 3 |
| 3 | 9 | 6 | 0 |
| 3 | 10 | 0 | 0 |
| 4 | 1 | 0 | 0 |
| 4 | 2 | 0 | 0 |
| 4 | 3 | 0 | 0 |
| 4 | 4 | 0 | 0 |
| 4 | 5 | 0 | 1 |
| 4 | 6 | 7 | 5 |
| 4 | 7 | 5 | 5 |
| 4 | 8 | 1 | 1 |
| 4 | 9 | 0 | 0 |
| 4 | 10 | 0 | 0 |
| 5 | 1 | 0 | 0 |
| 5 | 2 | 0 | 0 |
| 5 | 3 | 0 | 1 |
| 5 | 4 | 0 | 1 |
| 5 | 5 | 0 | 0 |
| 5 | 6 | 1 | 1 |
| 5 | 7 | 9 | 4 |
| 5 | 8 | 0 | 4 |
| 5 | 9 | 2 | 0 |
| 5 | 10 | 0 | 0 |
| 6 | 1 | 0 | 0 |
| 6 | 2 | 0 | 0 |
| 6 | 3 | 0 | 0 |
| 6 | 4 | 0 | 0 |
| 6 | 5 | 0 | 0 |
| 6 | 6 | 0 | 0 |
| 6 | 7 | 3 | 3 |
| 6 | 8 | 9 | 14 |
| 6 | 9 | 1 | 3 |
| 6 | 10 | 0 | 4 |
| 7 | 1 | 0 | 0 |
| 7 | 2 | 0 | 0 |
| 7 | 3 | 1 | 0 |
| 7 | 4 | 0 | 0 |
| 7 | 5 | 0 | 0 |
| 7 | 6 | 0 | 0 |
| 7 | 7 | 0 | 1 |
| 7 | 8 | 8 | 3 |
| 7 | 9 | 1 | 6 |
| 7 | 10 | 0 | 0 |
| 8 | 1 | 0 | 0 |
| 8 | 2 | 0 | 0 |
| 8 | 3 | 0 | 0 |
| 8 | 4 | 0 | 0 |
| 8 | 5 | 0 | 0 |
| 8 | 6 | 0 | 0 |
| 8 | 7 | 0 | 0 |
| 8 | 8 | 1 | 0 |
| 8 | 9 | 0 | 3 |
| 8 | 10 | 0 | 0 |
| 9 | 1 | 0 | 0 |
| 9 | 2 | 0 | 0 |
| 9 | 3 | 0 | 0 |
| 9 | 4 | 0 | 0 |
| 9 | 5 | 0 | 0 |
| 9 | 6 | 0 | 0 |
| 9 | 7 | 0 | 0 |
| 9 | 8 | 0 | 0 |
| 9 | 9 | 0 | 0 |
| 9 | 10 | 0 | 0 |
| 10 | 1 | 0 | 0 |
| 10 | 2 | 0 | 0 |
| 10 | 3 | 0 | 0 |
| 10 | 4 | 0 | 0 |
| 10 | 5 | 0 | 0 |
| 10 | 6 | 0 | 0 |
| 10 | 7 | 0 | 0 |
| 10 | 8 | 0 | 0 |
| 10 | 9 | 0 | 0 |
| 10 | 10 | 0 | 0 |

**S3 Table.** The serotyping IFA endpoint titer data. The counts of CKDu patient and healthy serum sample end-point titers against ANJZV and LNKV rGn antigens.
