# Supplementary material for: Lanka virus, a Mus booduga-borne orthohantavirus infection-associated febrile illness in Sri Lanka
Source: PLoS Negl Trop Dis. 2025 Jun 11;19(6):e0013169. doi: 10.1371/journal.pntd.0013169 (PMC12193775; doi:10.1371/journal.pntd.0013169)
Supplement: S1 Text — (DOCX) [file pntd.0013169.s006.docx]

**Supporting Information File**

**Supporting Methods**

Methods for alternative neutralization assay

**Preparation of pseudotype viruses.**

Pseudotype viruses were prepared as previously described (36). Briefly, for the preparation of pseudotype viruses bearing LNKV and ANJZV GPs, a recombinant vesicular stomatitis virus (VSV) derived from a full-length cDNA clone of the VSV genome (Indiana serotype) in which the coding region of the G protein was replaced by the coding region of the GFP gene and the G protein was expressed in trans designated as VSV△G*G was used as the stock virus. At 36 hours after transfection of 293T cells with pCLNK-M and pCANJZ-M, the cells were infected with VSV△G*G at a multiplicity of infection of 1 for 1 hour at room temperature. The 293T cell monolayer was then washed with 1% heat-inactivated FCS-PBS three times, and the culture medium was added. After 48 hours of incubation at 37°C in a CO_2_ incubator, the culture supernatant was clarified by low-speed centrifugation and stored at -80°C. The pseudotype viruses bearing LNKV and ANJZV GPs were designated as VSV△G*LNKV and VSV△G*ANJZV, respectively.

**Titration of pseudotype viruses.**

For pseudotype virus titration, Vero E6 cell monolayers grown on 96-well plates were infected with 50 µL of serially diluted virus stock. After a 1-hour adsorption period, the inoculum was removed, fresh culture medium was added, and the cells were incubated at 37°C in a CO_2_ incubator. At 16 hours post-infection, the cells were fixed with 2% paraformaldehyde in PBS for 10 min at room temperature, washed with distilled water, and air dried. GFP-expressing cells were counted under a fluorescence microscope. Since pseudotype VSVs are unable to produce infectious progeny virus, the numbers of GFP-positive cells were regarded as infectious units (IU).

**Neutralization of VSV pseudotypes.**

A total of 30 µL of medium containing 100 IU of prepared VSV pseudotypes was incubated with an equal volume of serially diluted rodent sera for 1 h at 37°C. Then, 50 µL of the mixture was inoculated onto Vero E6 cell monolayers in 96-well tissue culture plates. After adsorption for 1 hour, the mixture was replaced with Eagle minimal essential medium. After a 20-hour incubation period, the cells were fixed with 2% paraformaldehyde for 10 min, washed with distilled water, and air dried. Cells infected with VSV pseudotypes were examined and counted based on GFP expression under a fluorescence microscope. 80% reduction of GFP-expressing cells was considered for the neutralizing titer determination.
